# Downregulation of E-Cadherin enhances proliferation of head and neck cancer through transcriptional regulation of EGFR

**DOI:** 10.1186/1476-4598-10-116

**Published:** 2011-09-22

**Authors:** Dongsheng Wang, Ling Su, Donghai Huang, Hongzheng Zhang, Dong M Shin, Zhuo G Chen

**Affiliations:** 1 Department of Hematology and Medical Oncology, Winship Cancer Institute, Emory University School of Medicine, Atlanta, GA, USA

## Abstract

**Background:**

Epidermal growth factor receptor (EGFR) has been reported to downregulate E-cadherin (E-cad); however, whether the downregulation of E-cad has any effect on EGFR expression has not been elucidated. Our previous studies have found an inverse correlation between EGFR and E-cad expression in tissue specimens of squamous cell carcinoma of the head and neck (SCCHN). To understand the biological mechanisms underlying this clinical observation, we knocked down E-cad expression utilizing E-cad siRNA in four SCCHN cell lines.

**Results:**

It was observed that downregulation of E-cad upregulated EGFR expression compared with control siRNA-transfected cells after 72 hours. Cellular membrane localization of EGFR was also increased. Consequently, downstream signaling molecules of the EGFR signaling pathway, p-AKT, and p-ERK, were increased at 72 hours after the transfection with E-cad siRNA. Reverse transcriptase-polymerase chain reaction (RT-PCR) showed EGFR mRNA was upregulated by E-cad siRNA as early as 24 hours. In addition, RT-PCR revealed this upregulation was due to the increase of EGFR mRNA stability, but not protein stability. Sulforhodamine B (SRB) assay indicated growth of E-cad knocked down cells was enhanced up to 2-fold more than that of control siRNA-transfected cells at 72-hours post-transfection. The effect of E-cad reduction on cell proliferation was blocked by treating the E-cad siRNA-transfected cells with 1 μM of the EGFR-specific tyrosine kinase inhibitor erlotinib.

**Conclusion:**

Our results suggest for the first time that reduction of E-cad results in upregulation of EGFR transcriptionally. It also suggests that loss of E-cad may induce proliferation of SCCHN by activating EGFR and its downstream signaling pathways.

## Background

Head and neck cancer (HNC) is the sixth most common cancer and is responsible for almost 200,000 deaths around the world each year [1,2]. There were an estimated 48,010 new cases of HNC and 10,260 deaths in the U.S alone in 2009 [3]. HNC presents as 90% squamous cell carcinoma (SCC) and is a highly heterogeneous disease. Both locoregional recurrences and lymph node metastasis (LNM) are associated with a poor prognosis. Despite advances in understanding the molecular mechanisms of HNC along with improved diagnosis, the 5-year survival rate has been virtually unchanged in the past 30 years, remaining at less than 50% for patients with a single ipsilateral lymph node metastasis and less than 25% for patients with bilateral metastasis. Therefore, better understanding of the biological behavior of this disease could help to predict and guide treatment of HNC.

Epidermal growth factor receptor (EGFR) is a 170 kDa transmembrane protein with intrinsic tyrosine kinase activity that regulates cell growth in response to binding of its ligands, including epidermal growth factor (EGF) and transforming growth factor-α (TGF-α). EGFR overexpression has been documented extensively in a wide variety of malignant tumors, including squamous cell carcinoma of the head and neck (SCCHN) [4-11]. Overexpression of EGFR and its ligand TGF-α is observed in 80 to 90% of SCCHN specimens [7,8,12-14]. Several studies have demonstrated that EGFR overexpression correlates with reduced disease-free and overall survival [6,9,10,12]. Therefore, many strategies including using specific tyrosine kinase inhibitors (TKI) and monoclonal antibodies to target EGFR have been developed for treatment of SCCHN.

E-cadherin (E-cad) is a cell-cell adhesion transmembrane molecule. It plays important roles not only in cell adhesion and morphogenesis, but also in cellular signal transduction in collaboration with EGFR/ERK and c-Src-mediated pathways. In addition, loss of E-cad results in the translocation of β-catenin into the nucleus, permitting direct and indirect regulation of transcription. It has also been shown that loss of E-cad is involved in epithelial-mesenchymal transition (EMT) which is the hallmark for cancer metastasis [15]. E-cad expression in SCCHN tissue specimens has been reported in several studies. Together, these studies have demonstrated the essential roles of EGFR and E-cad in SCCHN cancer development and progress.

Previous studies have indicated there are cross-talks between the E-cad and EGFR pathways regulating the growth of various types of cancer. It has been demonstrated that activation of EGFR reduced E-cad levels through the E-cad suppresser gene TWIST [16]. E-cad has been reported to bind to EGFR via the extracellular domain of both proteins, and as such inhibit its activation. Lugo-Martínez *et al *have shown that activation of EGFR was detected in detached enterocytes before the disappearance of E-cad, and that endocytosis of E-cad depended on the tyrosine-kinase activity of EGFR [17]. These results indicate that a mutual regulation exists between E-cad and EGFR. Although this has been studied intensely, it remains unknown whether the reduction of E-cad has any regulatory effect on EGFR in terms of both expression and function.

Our own studies have shed light on the expression and cellular localization of EGFR and E-cad in both tumor specimens and SCCHN cell lines [18,19]. Three patterns in the tumor samples were observed, in which 48% showed overexpression of EGFR and reduced expression of E-cad. SCCHN patients with this expression pattern also demonstrated shorter disease-free and overall survival than the patients with the two other patterns [18]. To understand the biology behind this observation and its implication for SCCHN, we used siRNA to reduce E-cad expression to determine whether downregulation of E-cad has any effect on EGFR expression and function, which may consequently accelerate SCCHN cell proliferation.

## Methods

### Cell Culture

Four E-cad highly-expressing SCCHN cell lines were used in these studies. Tu686 and 686LN were established from a primary base of tongue SCC and its lymph node metastasis, respectively [20]. Tu212 was established from a primary hypopharyngeal SCC [21]. UPCI-37A was established from SCC in the larynx (epiglottis) at the University of Pittsburgh Cancer Institute [22]. The cell lines were maintained as monolayer cultures in Dulbecco's modified Eagle's medium (DMEM)/F12 medium (1:1) supplemented with 10% fetal bovine serum (FBS) at 37°C in a humidified atmosphere with 5% CO_2._

### Reduction of E-cad by siRNA

Cells were seeded at a concentration of 1.5 × 10^5^/well in 12-well cell culture plates 12 hrs before transfection. Both E-cad siRNA (siGENOME SMART pool, Cat# M-003877-02) and non-targeted control siRNA (siCONTROL, Cat# D-001210-01) (Thermo Scientific Dharmacon, Chicago, IL) were transfected using HiPerFect transfection reagent (Qiagen, Valencia, CA) according to the manufacturer's instruction. The effects on E-cad reduction of the transfection were evaluated by Western blot and flow cytometry analysis as described below.

### Western Blot Analysis

Cells were washed twice with phosphate buffered saline (PBS) before being lysed on ice for 30 min with lysis buffer containing 50 mmol/L HEPES buffer, 150 mmol/L NaCl, 1 mmol/L EDTA (pH 8.0), 1 mmol/L EGTA(pH8.0), 1% IGEPAL CA-630, 0.5% Triton X-100, 10 mmol/L NaF, 2 mmol/L Na_3_VO_4_, 10 mmol/L β-glycerophosphate and 1% Protease Inhibitor Cocktail (Sigma-Aldrich, St Louis, MO). The lysate was centrifuged at 16,000 g at 4°C for 15 min. Fifty micrograms of total protein for each sample were separated by 10% SDS-PAGE and transferred onto a polyvinylidene difluoride membrane (Bio-Rad Labs, Hercules, CA), and the desired proteins were probed with corresponding antibodies. Mouse anti-E-cad (clone G-10, 1:1000 dilution) and rabbit anti-EGFR (clone 1005, 1:500 dilution) antibodies were purchased from Santa Cruz Biotechnology. Phospho-p42/44 MAPK (Thr202/Tyr204) and phospho-AKT (Ser473) antibodies were purchased from Cell Signaling Technology. Antibody for phospho-EGFR (Tyr1173, 1:500 dilution) was purchased from Upstate. GAPDH (1:3000 dilution, Trevigen, Inc., Gaithersburg, MD) was used as an internal control. HRP-conjugated secondary antibodies were anti-Rabbit IgG (H+L) and anti-Mouse IgG (H+L) (Promega, Madison, WI). Bound antibody was detected using the SuperSignal West Pico Chemoluminescence system (Pierce, Inc., Rockford, IL).

### Flow Cytometric Analysis

To quantify EGFR expression on the cell membrane, flow cytometric analysis of cell surface staining was performed. After dissociation with Cell Stripper™ (Mediatech, Herndon, VA), 1 × 10^6 ^cells were incubated with monoclonal mouse-anti-EGFR (R-1, 1:200 dilution, Santa Cruz) for 45 min at room temperature. Mouse IgG was used as an isotype control. After washing, the cells were incubated with Alexa Fluor^® ^488 goat anti-mouse IgG (H+L) highly cross-absorbed antibody (1:200 dilution, Molecular Probe). Cells were resuspended in 1 ml 2% BSA to run flow cytometry analysis with FACScan (BD). Data were analyzed by the software FlowJo (Ashland, OH).

### RT-PCR and Real-Time PCR

Total RNA of siRNA-transfected cells was isolated with Trizol reagent (Invitrogen, Carlsbad, CA) and reverse transcription was performed by using iScript™ cDNA Synthesis Kit (Bio-Rad) according to the manufacturer's protocol. PCR was performed using Taq DNA Polymerase High Fidelity (Invitrogen) with the primers indicated below. Forward primer for EGFR: 5'-TGTTT GGGAC CTCCG GTCAG-3' and reverse primer for EGFR: 5'-GGCAG GTCTT GACGC AGTGG-3'. GAPDH was also amplified as the internal control with the following primers: forward: 5'-ATGTT CGTCA TGGGT GTGAA CCA-3' and reverse: 5'-TGGCA GGTTT TTCTA GACG GCAG-3'. All samples were first denatured at 94°C (5 min) and the PCR reaction then allowed to proceed for 25 (GAPDH) and 30 (EGFR) amplification cycles: denaturation (45 sec, 94°C), annealing (1 min at 57°C) and extension (1 min at 72°C). Then, a final extension step of 10 min at 72°C was performed. EGFR and GAPDH mRNA level was quantified using UVP BioImaging System, (Upland CA) to measure the density of specific cDNA band.

For real-time PCR, equal amounts of total mRNA from E-cad knock-down cells and control cells were used to amplify cDNA. EGFR and GAPDH cDNAs were amplified with iQ SYGR Green Supermix (Bio-Rad, Hercules CA) using the same primers as described above. The reaction mixture consisted of 0.5 μl of cDNA, 25 μl of iQ SYGR Green Supermix, 0.2 μM of target primers in a total volume of 50 μl. Amplification was carried out at 10 min at 95°C for polymerase activation, and 35 cycles of 95°C for 15 s (denaturation) and 56°C for 1 min (annealing and extension) on the IQ5 real-time detection system (Bio-Rad). The amount of EGFR mRNA was normalized to human GAPDH as an internal control. Experiments were repeated 3 times. Error bars represent standard deviation.

### EGFR mRNA Stability Assay

A set of siRNA transfected cells were re-seeded in a 12-well plate 24 hrs after the transfection. After settling, the cells were exposed to actinomycin-D (Sigma-Aldrich) at 5 μg/ml. RNA was harvested at 0, 4 hrs, 8 hrs, and 24 hrs. The levels of EGFR mRNA were determined by RT-PCR as described above.

### EGFR Protein Stability Assay

A set of siRNA transfected cells were re-seeded in a 12-well plate 24 hrs after the transfection. After settling, the cells were exposed to cycloheximide (CHX) (Sigma-Aldrich) at 10 μg/ml. RNA was harvested at 0, 1 hr, 3 hrs, and 24 hrs. The levels of EGFR protein were determined by Western blot analysis as described above.

### Cell Growth Assay (SRB assay)

Sulforhodamine B (SRB) assay was used for cell growth determination. siRNA-transfected cells were re-seeded in a 96-well plate 24 hrs after the transfection at a density of 5 × 10^3 ^cells/well. Cells were fixed with 10% trichloroacetic acid after another 24, 48, or 72 hrs of culture. Cells then were washed 5 times with distilled and de-ionized water. After air drying, 50 μl SRB was added to the cells and incubated for 10 min. Cells were then washed with 1% acetic acid 5 times. After air drying, 10 mM Tris solution (pH 10) was added to dissolve the bound dye. The cell growth was assessed by optical density (OD) determination at 510 nm using a microplate reader. For the TKI study, 1 μM erlotinib was added 24 hrs after cells were transfected with siRNA. SRB assay was carried out 48 and 72 hrs after erlotinib treatment.

## Results

### Downregulation of E-cad enhanced EGFR expression mainly through stabilization of EGFR mRNA

**Figure 1:**
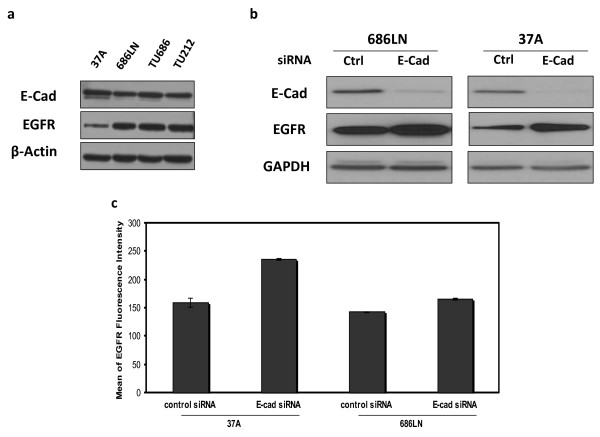
**Reduction of E-cad by siRNA increased the levels of total EGFR and cell surface EGFR in both 686LN and PCI-37A cells**. (a) Basal E-cad and EGFR expression in 4 SCCHN cell lines. (b) Both E-cad siRNA and non-targeted control siRNA were transfected using HiPerFect transfection reagent. The effects of E-cad reduction by siRNA transfection on EGFR expression at the protein level were evaluated by Western blot. At 72 hours after the transfection, EGFR protein levels were clearly increased in both 686LN and PCI-37A cells. (c). To quantify EGFR expression on the cell membrane, flow cytometric analysis of cell surface staining was performed. The cells transfected with siRNA (48 hrs after transfection) were incubated with monoclonal mouse-anti-EGFR antibody (Santa Cruz). The level of EGFR protein on the cell surface was examined by flow cytometry analysis using FACScan (BD). Data were analyzed by the software FlowJo (Ashland, OH). Data indicate that in both 686LN and PCI-37A cells, the level of cell surface EGFR protein was significantly increased upon reduction of E-cad based on three measurements (p < 0.001 for both cell lines). Error bars represent standard deviation.

**Figure 2:**
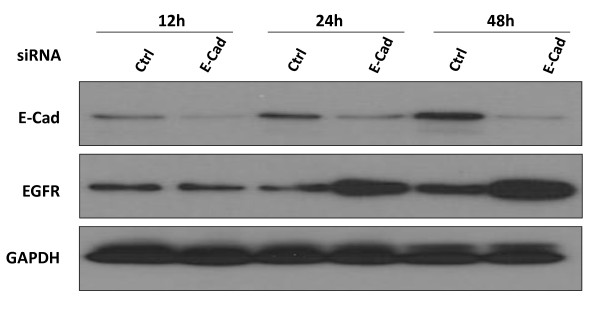
**EGFR upregulation can be detected as early as 24 hours in E-cad siRNA transfected cells**. Cell lysate from PCI-37A cells was collected at 12, 24, and 48 hours after transfection with E-cad and control siRNAs. 50 μg protein was loaded for SDS-PAGE, and Western blot analysis was performed using EGFR antibody. The result showed EGFR protein was upregulated as early as 24 hours after siRNA was transfected.

**Figure 3:**
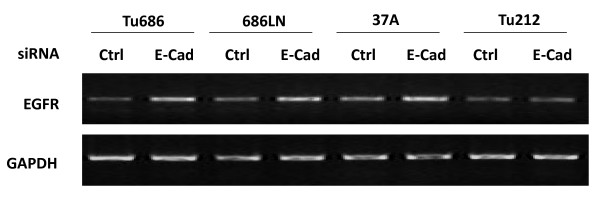
**Reduction of E-cad by siRNA increased EGFR mRNA in SCCHN cell lines**. Both E-cad siRNA and non-targeted control siRNA were transfected using HiPerFect transfection reagent. The EGFR mRNA level was evaluated 24 h after the transfection by RT-PCR. RT-PCR on GAPDH was performed at the same time as a control. Three out of four cell lines (Tu686, 686LN, PCI-37A) showed increased expression of EGFR mRNA.

**Figure 4:**
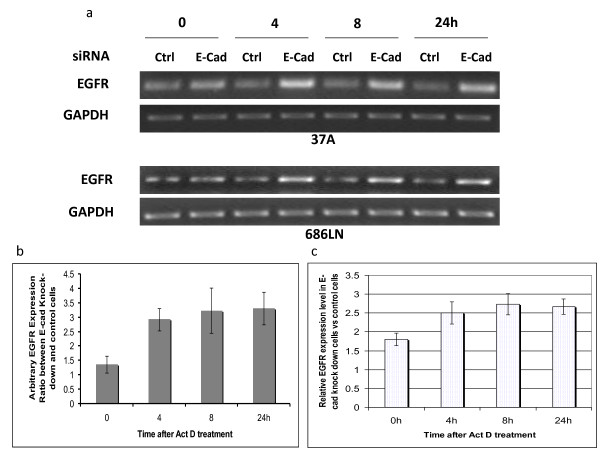
**Knockdown of E-cad increased EGFR expression by enhancing its mRNA stability**. (a) 24 hours after transfection of PCI-37A and 686LN cells with siRNA, cells were treated with actinomycin-D (Sigma-Aldrich) at 5 μg/ml for 0, 4, 8 and 24 hours. RT-PCR showed that the EGFR mRNA level remained higher in E-cad knockdown cells than in the control cells even after mRNA synthesis was stopped by actinomycin-D for 24 hours. (b) EGFR and GAPDH mRNA level as PCR products were measured using UVP BioImaging System Representative. The ratios of EGFR mRNA levels in E-cad knockdown cells (PCI-37A) against that in control cells at different time points were determined after being normalized to GAPDH. (c) Real-time RT-PCR on PCI-37A cells confirmed the ratios of EGFR mRNA level in E-cad knockdown cells against the control cells at different time points. These results indicate that EGFR mRNA stability was enhanced in E-cad knockdown cells. The experiments were repeated 3 times. Error bars represent standard deviation.

Expression levels of EGFR and E-cad were initially examined in four SCCHN cell lines: Tu686, 686LN, Tu212, and PCI-37A (Figure 1a). To determine whether the reduction of E-cad has any effect on EGFR expression level, and the mechanism of the possible regulation of EGFR by E-cad, we transfected two SCCHN cell lines, 686LN and PCI-37A with siRNA against E-cad. Western blot was performed to measure the change in EGFR protein level. It was shown that reduction of E-cad by siRNA increased the total protein levels of EGFR. The fold changes in total EGFR as compared with the control were 1.85 ± 0.2 and 2.1 ± 0.2 for 686LN and PCI-37A, respectively, based on three experiments (Figure 1b). The control siRNA did not have any effect on E-cad expression (see additional file 1). Upregulation of EGFR expression was also observed using a shRNA strategy targeting a different sequence to knock down E-cad (see additional file 2). At the same time, the amount of EGFR on the cellular membrane was also enhanced (Figure 1c). The fold changes in the level of cell surface EGFR as compared with the control were 1.22 ± 0.03 and 1.48 ± 0.07 for 686LN and PCI-37A cells, respectively, suggesting that increased EGFR on the surface after E-cad knockdown results mainly from the increase in total EGFR expression. EGFR upregulation was found as early as 24 hours after the siRNA treatment (Figure 2). RT-PCR was further performed to measure the change in EGFR mRNA levels. As shown in Figure 3, the mRNA level was upregulated in 3 out of 4 cell lines. To demonstrate the mechanism underlying the upregulation of EGFR by E-cad reduction, both PCI-37A and 686LN cells were treated with actinomycin D or cycloheximide (CHX). As shown in Figure 4a and 4b, the ratio of EGFR mRNA level between E-cad knockdown and the control cells increased from 0 to 24 h after actinomycin D treatment (1.3 ± 03, 2.9 ± 0.4, 3.2 ± 0.8, and 3.3 ± 0.6 after 0, 4, 8, and 24 hours, respectively, in PCI-37A cells). This result was confirmed by quantitative real-time PCR which showed that the ratios of EGFR mRNA level in E-cad knockdown cells against the control cells were 1.8 ± 0.16, 2.5 ± 0.30, 2.7 ± 0.29, and 2.6 ± 0.21 at different time points: 0, 4, 8, and 24 hours, respectively (Figure 4c), suggesting that EGFR mRNA stability was increased in E-cad knockdown cells compared with the control cells. The same result was also observed when shRNA was used to knock down E-cad expression (see additional file 3).

**Figure 5:**
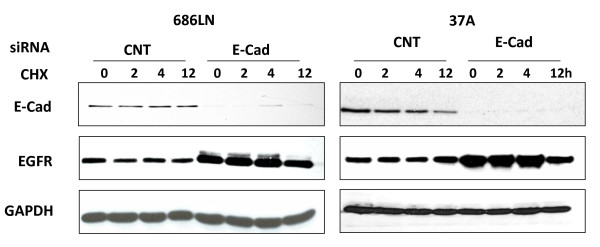
**Knockdown of E-cad did not increase EGFR protein stability**. 24 hours after PCI-37A and 686LN cells were transfected with siRNA, cells were treated with cycloheximide (CHX) (Sigma-Aldrich) at 10 g/ml for 0, 2, 4 and 12 hrs. The EGFR protein level remained unchanged for up to 4 hours, and then declined after 12 hours of treatment with CHX in the tested SCCHN cell lines with E-cad knockdown. The experiment was repeated once. Activity of CHX was confirmed with a protein known to be regulated at the transcriptional level by Western analysis.

On the other hand, EGFR protein stability was not changed by CHX treatment for up to 4 hours (28 hours after addition of E-cad siRNA), but was affected in the knock-down cells at 12 hours after the treatment with CHX (36 hours after addition of E-cad siRNA; Figure 5). This suggests that the overall effect of loss E-cad on total EGFR may be a balance between the two contradictory consequences, stabilizing the mRNA, but degrading protein.

### Elevated EGFR protein by reduction of E-cad resulted in activation of EGFR-mediated signaling pathways

**Figure 6:**
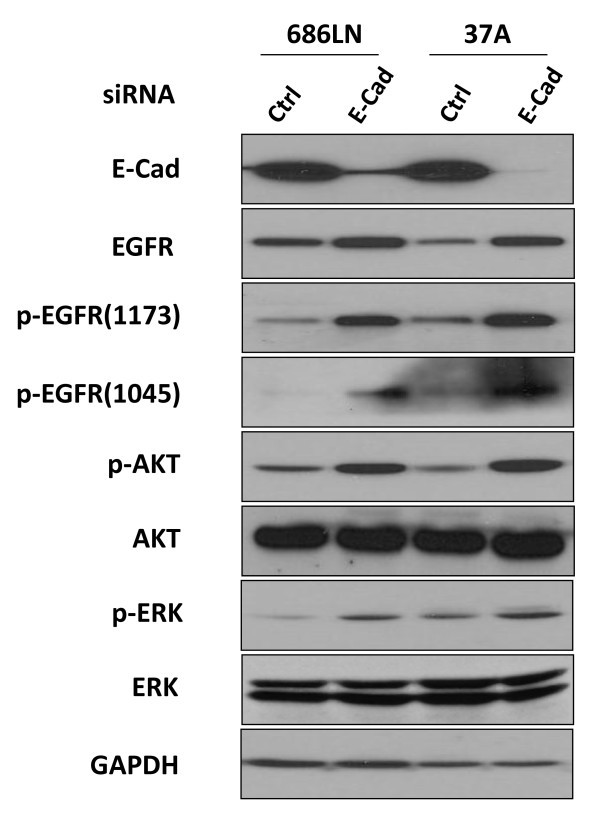
**Elevated EGFR protein by reduction of E-cad resulted in activation of EGFR-mediated signaling pathways**. Levels of phosphorylated AKT, phosphorylated ERK and phosphorylated EGFR were analyzed by Western blot. In both 686LN and PCI-37A cells, enhanced EGFR phosphorylation was observed after transfection of E-cad siRNA. Phosphorylated AKT and phosphorylated ERK levels were also increased without alteration of the total protein levels. These results indicate that E-cad knockdown activated the EGFR-mediated signaling pathway. Data presented are one result of 3 tests.

Our western blot results show that as E-cad is knocked down, the EGFR phosphorylation level at y1045 and y1173 increases in proportion to the increase in protein level (Figure 6). The increase in phosphorylation of EGFR can elicit activation of downstream signaling through several proteins. The involvement of AKT and ERK in EGFR-dependent phosphorylation cascades has long been recognized. To assess the role of the loss of E-cad in the EGFR signaling pathway, Western blot was carried out to analyze the alteration of these downstream target proteins of EGFR. Western blot results showed that both the phosphorylation levels of AKT and ERK were significantly increased by the reduction of E-cad (Figure 6), but the total levels of both AKT and ERK were not changed, suggesting that reduction of E-cad not only increased the protein and phosphorylation level of EGFR but also activated the downstream targets of EGFR. The same phenomena were observed using the shRNA strategy (see additional file 2).

### Downregulation of E-cad stimulated cell proliferation through activation of EGFR

**Figure 7:**
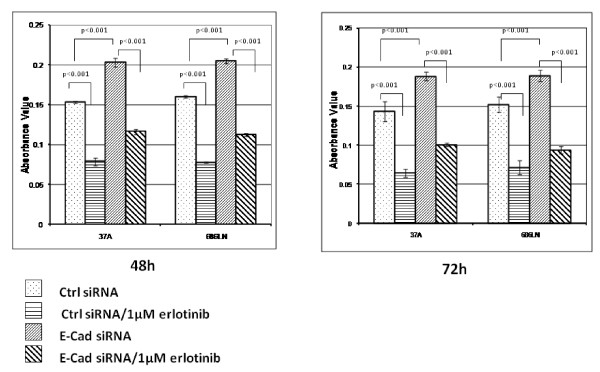
**Cell growth induced by E-cad knockdown can be inhibited by EGFR tyrosine kinase inhibitor**. Sulforhodamine B (SRB) assay was used for cell growth determination. E-cad siRNA-transfected PCI-37A and 686LN cells were re-seeded in a 96-well plate 24 hrs after the transfection. To check whether activation of EGFR contributed to cell proliferation, reseeded transfected cells were treated with 1 μM EGFR-specific tyrosine kinase inhibitor erlotinib for 48 or 72 hrs followed by fixation with 10% trichloroacetic acid and SRB assay. The OD at 510 nm was determined as absorbance value. The results showed that E-cad knockdown clearly induced cell growth, which, however, could be inhibited by treatment with 1 μM erlotinib. Data presented are 1 of 3 repeated independent experiments. Error bars represent standard deviation.

One of the results of the activation of EGFR and its downstream proteins is to initiate several signal transduction cascades and DNA synthesis followed by cell proliferation. To investigate the effect of reduction of E-cad on cell proliferation, we performed an SRB assay to assess cell growth after transfection with E-cad-specific siRNA. As shown in Figure 7, reduction of E-cad by siRNA increased the proliferation level of SCCHN cell lines by 1.25 to 1.5-fold in both PCI-37A and 686LN cells. To determine if the proliferation effect of E-cad reduction was EGFR dependent, we treated the siRNA-transfected cells with erlotinib, an EGFR-tyrosine kinase inhibitor (EGFR-TKI). Erlotinib clearly reduced the promotive effect of E-cad loss on cell proliferation.

## Discussion

EGFR overexpression and E-cad loss are the major characteristics of aggressive cancers [5,6,18,23,24]. It has been documented that these two characteristics are related and are identified as one of the major patterns in clinical tissue samples of SCCHN [18]. It has long been known that E-cad is involved in cancer progression. Loss of E-cad is related to cancer invasion and metastasis of SCCHN. However, how this loss of E-cad functions to promote cancer progression is still not completely elucidated. In this study, we demonstrate for the first time that loss of E-cad transcriptionally upregulates EGFR; the induced proliferation of SCCHN cells by loss of E-cad occurs at least partially through the activation of EGFR and its downstream signaling pathways.

EGFR is known to diminish E-cad expression by elevating MMP-9 activity. EGFR activation induces MMP-9 expression and activation, which in turn disrupts adhesion junction and protein level of E-cad [23]. On the other hand, previous studies have also shown that E-cad has the capacity to interact with EGFR functionally. Qian *et al *showed that E-cad could inhibit cellular responses to EGFR stimulation [25]. They observed that mitogenic responsiveness to EGF decreased as cells grew to confluence. This desensitization could be overcome by adding antibodies that block E-cad function. They also showed reduction of E-cad increased both EGFR autophosphorylation and EGF-induced DNA synthesis. Perrais *et al *further demonstrated that E-cad homophilic ligation inhibited serum-stimulated cell proliferation by preventing E-cad binding to β-catenin [26]. They also demonstrated that E-cad ligation inhibited EGF-induced cell proliferation. E-cad homophilic ligation disrupted the ability of EGFR to activate DNA synthesis. Although these findings shed significant light on the mutual regulation of EGFR and E-cad, none have addressed whether the loss of E-cad, which is one of the most important features of EMT, has any effect on EGFR expression.

To demonstrate whether E-cad loss has any effect on EGFR expression and function, we knocked down E-cad expression by siRNA in four SCCHN cell lines. We then checked both mRNA and protein levels of EGFR in these transfected cells. Our results showed that both mRNA and protein levels of EGFR were upregulated when E-cad was knocked down. We further found that E-cad loss upregulated EGFR mRNA level by increasing its mRNA stability. Most likely, loss of E-cad affects EGFR mRNA stability indirectly since upregulation of EGFR was observed 24 hours after applying E-cad siRNA, which deserves further investigation. Currently, we cannot rule out whether loss of E-cad may also enhance EGFR transcription through upregulating transcription factors. There was no direct interaction observed between EGFR and E-cad in the tested cell lines by immunopreciptation (data not shown). It is still possible that ablation of E-cad stimulates EGFR expression through other proteins. Another mechanism by which high level EGFR expression could be sustained is through increased protein stability; however, we did not obtain any evidence in this regard.

We investigated whether the EGFR-mediated signaling pathway is affected by E-cad-mediated regulation. After E-cad was knocked down, the cellular membrane localization of EGFR was increased in addition to total EGFR protein, which prepares EGFR to be ready to respond to stimuli by EGFR ligands. This result suggests that E-cad loss could not only increase EGFR expression but also could have functional effects on the EGFR signaling pathway, but current experiments cannot prove whether the upregulation of EGFR expression is solely responsible for the observed activation of EGFR signaling. Our Western blot analysis showed that downstream signaling molecules of EGFR, p-AKT, and p-ERK, were increased at 72 hours after treatment with E-cad siRNA without a change in their total protein levels. AKT and ERK are the major signal mediators downstream of the EGFR pathway. The EGFR-Ras-Raf-MEK-ERK signaling pathway has been the subject of intense research and pharmaceutical scrutiny to identify novel target-based approaches for cancer treatment (24). AKT, which affects tumor cell motility and invasiveness, is also part of the EGFR-associated signaling network. Our results indicate that E-cad is probably involved in regulation of both EGFR-ERK and EGFR-AKT pathways, resulting in SCCHN cancer cell proliferation.

As speculated, the SRB assay showed that the growth rate of E-cad knockdown cells increased up to 2-fold more than that of control siRNA-transfected cells after 96 hours. The effect of E-cad reduction on cell proliferation was blocked by treating the E-cad siRNA-transfected cells with 1 μM of the EGFR-specific TKI erlotinib. These results support that E-cad loss has a significant effect on EGFR function as well as expression in SCCHN. They also indicate that the effect of E-cad knockdown on cell proliferation was at least partly dependent on EGFR activation.

EMT has been extensively studied because of its essential role in cancer metastasis. Loss of E-cad is a hallmark of EMT. Lo *et al *reported that EGFR activation by EGF led to EMT, an early event in carcinogenesis, and loss of E-cad by activation of TWIST through a STAT3-mediated pathway [16]. Snail is widely regarded as the suppressor of E-cad, the driving force behind EMT [27,28]. Activation of EGFR results in overexpression of Snail [29]. Our findings suggested that loss of E-cad induces EGFR expression. It is also possible that TWIST and Snail could be further activated in EMT as a result of overexpression of EGFR downstream of E-cad ablation, thus sustaining the EMT process. Additional studies certainly need to be done to address if this is the case. Taken together, our data clearly demonstrate that downregulating E-cad transcriptionally increases EGFR expression as well as its function. These results suggest that reduction of E-cad contribute to SCCHN cancer progression and malignancy not just by decreasing the strength of cellular adhesion, which results in an increase in cellular motility and cancer metastasis, but also by increasing EGFR expression and consequently its downstream signaling pathway, leading to enhanced cancer cell proliferation. Most importantly, this current work for the first time has demonstrated that loss of E-cad affects EGFR expression by increasing its mRNA stability.

## Abbreviations

EGFR: Epidermal growth factor receptor; E-cad: E-cadherin; SCCHN: squamous cell carcinoma of the head and neck; SRB: Sulforhodamine B assay; EMT: epithelial-mesenchymal transition; EGFR-TKI: EGFR-tyrosine kinase inhibitor; CHX: cycloheximide.

## Competing interests

The authors declare that they have no competing interests.

## Authors' contributions

DSW, LS, DH, HZ conducted experiments, data analysis, and experimental design. ZGC and DMS participated in experimental design and coordination. DSW and ZGC prepared the draft of the manuscript. All authors read and approved the final manuscript.

## Supplementary Material

Additional file 1 **Control siRNA does not affect E-cad expression in 4 cell lines**. After 686LN, TU212, TU686 and 37A cells were transfected with control siRNA for 24 hours, cell lysates from wild type cells and transfected cells were collected; 50 μg protein was loaded for Western blot to analyze the E-cad expression. β-actin expression was also detected as a loading control. Our data indicate that the control siRNA has no effect on E-cad expression. Data presented are one result of 3 separate experiments. Click here for file

Additional file 2 **Elevated EGFR protein by reduction of E-cad using pLKo.1 shRNA E-cad resulted in activation of EGFR-mediated signaling pathways**. Levels of phosphorylation of EGFR at y1173 and y1068, phosphorylated AKT, and phosphorylated ERK were analyzed by Western blot. In both 686LN and PCI-37A cells, enhanced EGFR phosphorylation was observed after transfection of pLKo.1 shRNA E-cad targeting sequence 5'-gcagaaattattgggctcttt-3' (Addgene Inc Cambridge, MA). Phosphorylated AKT and phosphorylated ERK levels were also increased without alteration of the total protein levels. Data presented are one representative out of three repeated experiments. Click here for file

Additional file 3 **Knockdown of E-cad with pLKO.1 shRNA E-cad increased EGFR expression by enhancing its mRNA stability**. After PCI-37A and 686LN cells were transfected with the pLKo.1 shRNA E-cad (see additional file 2) for 24 hours, cells were treated with actinomycin-D (Sigma-Aldrich) at 5 μg/ml for 0, 4, 8 and 24 hours. RT-PCR showed that the EGFR mRNA level remained higher in E-cad knockdown cells than in the pLKO.1-transfected control cells after mRNA synthesis was stopped by actinomycin. Click here for file
