# Supplementary figures and images for: Downregulation of E-Cadherin enhances proliferation of head and neck cancer through transcriptional regulation of EGFR
Source: Mol Cancer. 2011 Sep 22;10:116. doi: 10.1186/1476-4598-10-116 (PMC3192774; doi:10.1186/1476-4598-10-116)

## Slide 1
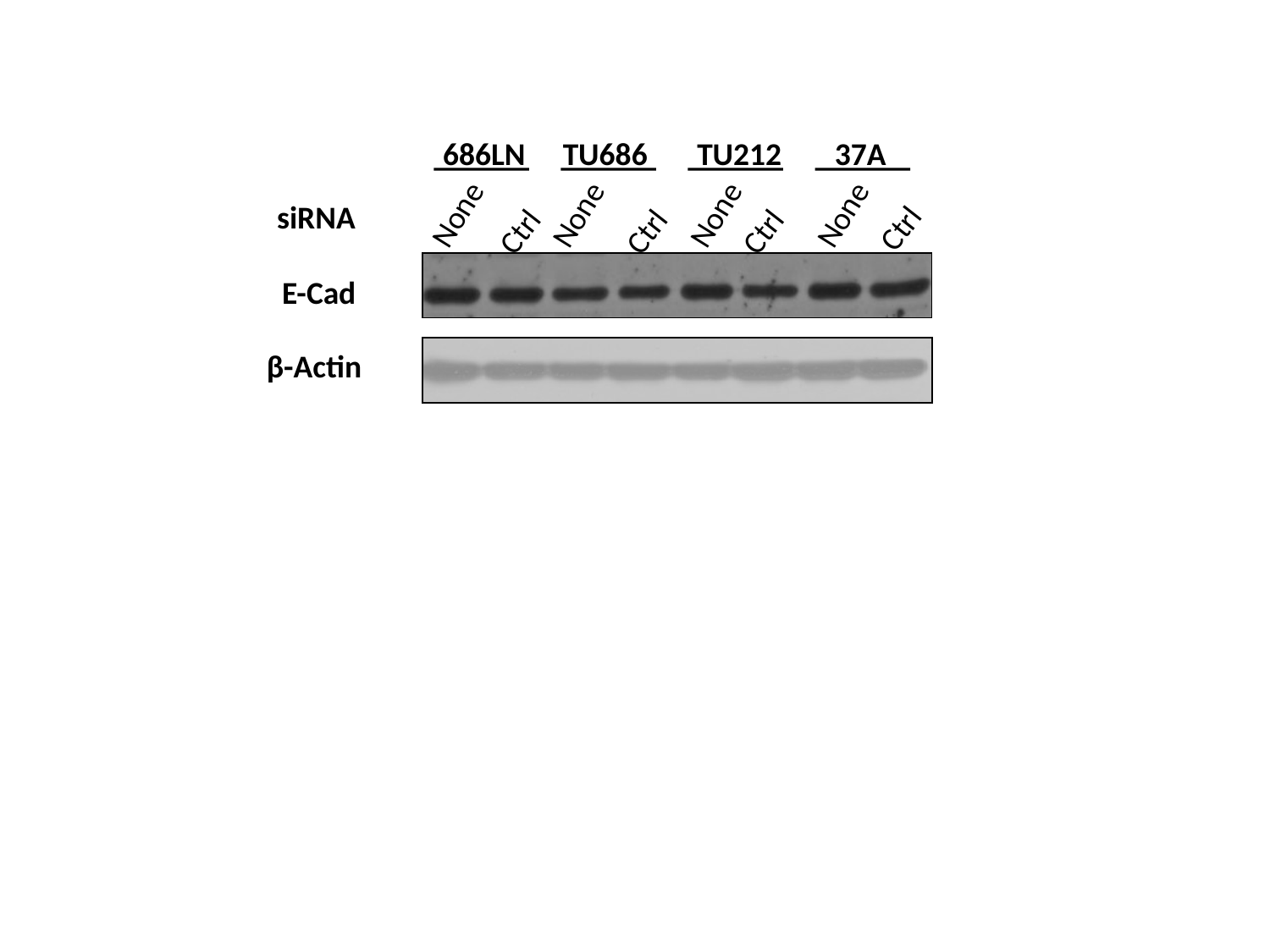

686LN
TU686
 TU212
 37A
None
None
None
None
siRNA
Ctrl
Ctrl
Ctrl
Ctrl
E-Cad
β-Actin

Supplement: Additional file 1 — Control siRNA does not affect E-cad expression in 4 cell lines. After 686LN, TU212, TU686 and 37A cells were transfected with control siRNA for 24 hours, cell lysates from wild type cells and transfected cells were collected; 50 μg protein was loaded for Western blot to analyze the E-cad expression. β-actin expression was also detected as a loading control. Our data indicate that the control siRNA has no effect on E-cad expression. Data presented are one result of 3 separate experiments. [file 1476-4598-10-116-S1.PPT]

## Slide 1
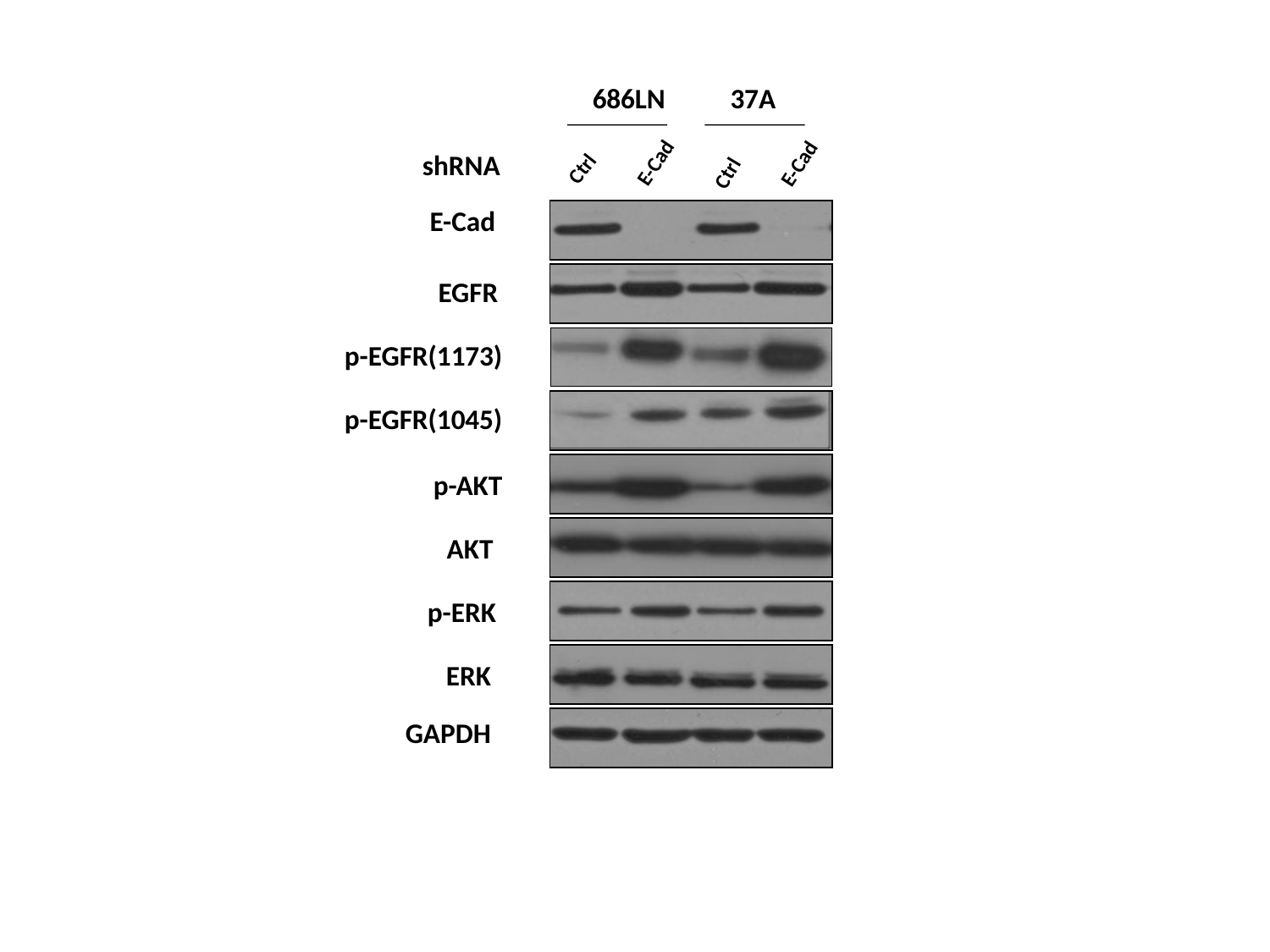

686LN
37A
E-Cad
E-Cad
Ctrl
Ctrl
shRNA
E-Cad
EGFR
p-EGFR(1173)
p-EGFR(1045)
p-AKT
AKT
p-ERK
ERK
GAPDH

Supplement: Additional file 2 — Elevated EGFR protein by reduction of E-cad using pLKo.1 shRNA E-cad resulted in activation of EGFR-mediated signaling pathways. Levels of phosphorylation of EGFR at y1173 and y1068, phosphorylated AKT, and phosphorylated ERK were analyzed by Western blot. In both 686LN and PCI-37A cells, enhanced EGFR phosphorylation was observed after transfection of pLKo.1 shRNA E-cad targeting sequence 5'-gcagaaattattgggctcttt-3' (Addgene Inc Cambridge, MA). Phosphorylated AKT and phosphorylated ERK levels were also increased without alteration of the total protein levels. Data presented are one representative out of three repeated experiments. [file 1476-4598-10-116-S2.PPT]

## Slide 1
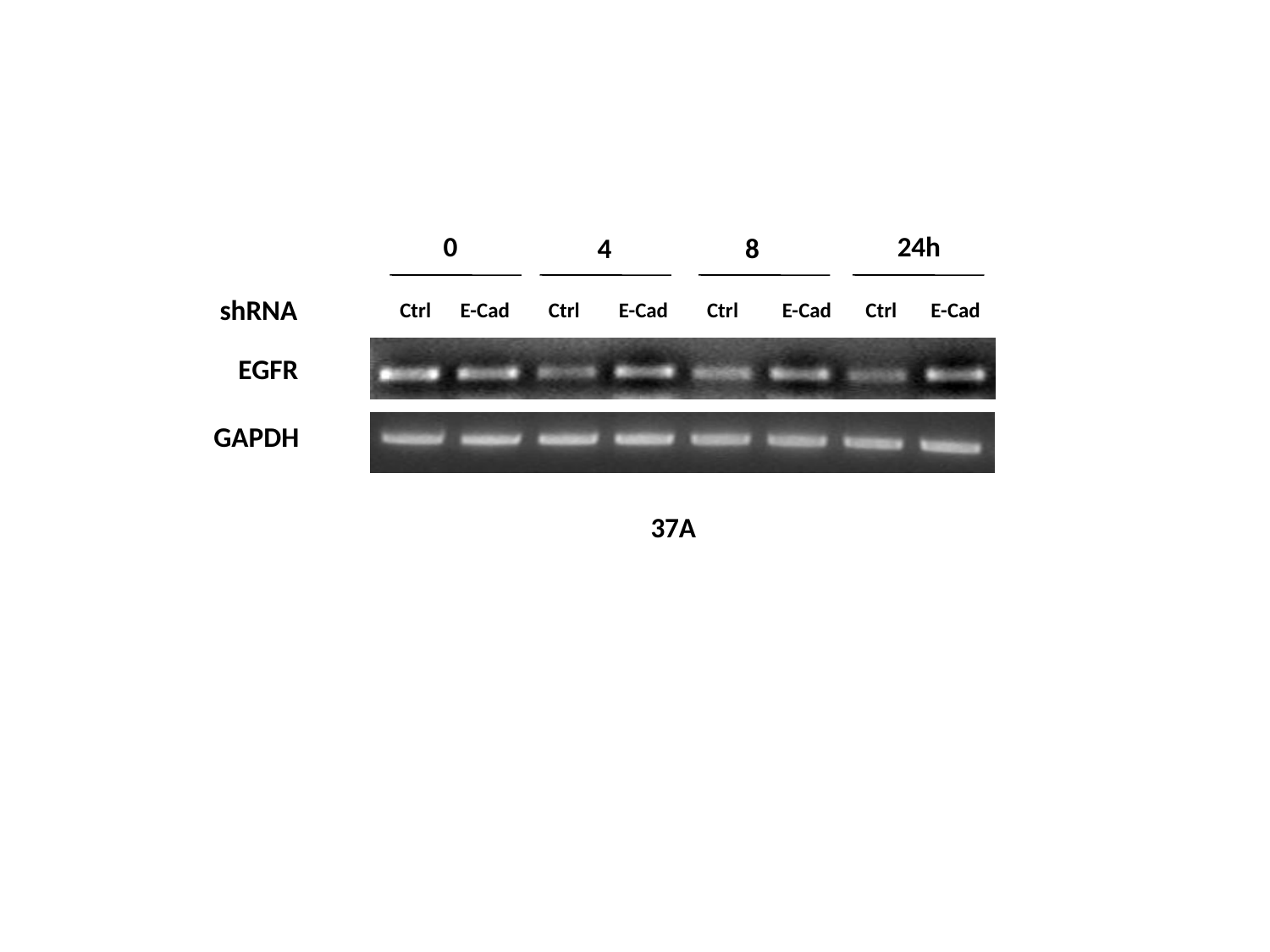

0
 24h
 4
 8
shRNA
 Ctrl E-Cad Ctrl E-Cad Ctrl E-Cad Ctrl E-Cad
EGFR
GAPDH
37A

Supplement: Additional file 3 — Knockdown of E-cad with pLKO.1 shRNA E-cad increased EGFR expression by enhancing its mRNA stability. After PCI-37A and 686LN cells were transfected with the pLKo.1 shRNA E-cad (see additional file 2) for 24 hours, cells were treated with actinomycin-D (Sigma-Aldrich) at 5 μg/ml for 0, 4, 8 and 24 hours. RT-PCR showed that the EGFR mRNA level remained higher in E-cad knockdown cells than in the pLKO.1-transfected control cells after mRNA synthesis was stopped by actinomycin. [file 1476-4598-10-116-S3.PPT]
